# AI-Powered Histology for Molecular Profiling in Brain Tumors: Toward Smart Diagnostics from Tissue

**DOI:** 10.3390/cancers18010009

**Published:** 2025-12-19

**Authors:** Maki Sakaguchi, Akihiko Yoshizawa, Kenta Masui, Tomoya Sakai, Takashi Komori

**Affiliations:** 1 Department of Diagnostic Pathology, Nara Medical University, Kashihara 634-8521, Japan; 2 Department of Integrative Neuroscience, Graduate School of Biomedical Sciences, Nagasaki University, Nagasaki 852-8523, Japan; 3 Institute of Integrated Science and Technology, Nagasaki University, Nagasaki 852-8521, Japan; 4 Department of Laboratory Medicine and Pathology, Tokyo Metropolitan Neurological Hospital, Tokyo 183-0042, Japan; 5 Department of Laboratory Medicine and Pathology, TMG Asaka Medical Center, Saitama 351-0023, Japan; 6 Department of Neurosurgery, Tokyo Women’s Medical University Hospital, Tokyo 162-8666, Japan

**Keywords:** artificial intelligence, multimodal AI, brain tumor, World Health Organization, molecular classification, intraoperative diagnosis, radiopathomics

## Abstract

The integration of molecular features into histopathological diagnoses has become central to the World Health Organization (WHO) classification of central nervous system (CNS) tumors, improving prognostic accuracy and supporting precision medicine. However, unequal access to molecular testing limits the universal application of integrated diagnosis. To address this, artificial intelligence (AI) models are being developed to predict molecular alterations directly from histological data. In gliomas, deep learning applied to whole-slide images (WSIs) of permanent sections achieves neuropathologist-level accuracy in predicting biomarkers such as IDH mutation and 1p/19q co-deletion, as well as in molecular subtype classification and outcome prediction. Recent advances extend these approaches to intraoperative cryosections, enabling real-time glioma grading, molecular prediction, and label-free tissue analysis using modalities such as stimulated Raman histology and domain-adaptive image translation. Beyond gliomas, AI-powered histology is being explored in other brain tumors, including morphology-based molecular classification of spinal cord ependymomas and intraoperative discrimination of gliomas from primary CNS lymphomas. This review summarizes current progress in AI-assisted molecular profiling prediction of brain tumors from tissue, highlighting opportunities for rapid, accurate, and globally accessible diagnostics. The integration of histology and computational methods holds promise for the development of smart AI-assisted neuro-oncology.

## Simple Summary

Artificial intelligence (AI) has rapidly entered the field of neuropathology, showing promise in the classification and molecular prediction of brain tumors. In particular, deep learning applied to digital histopathology has enabled accurate recognition of glioma subtypes, prediction of molecular alterations, and even intraoperative decision support. This review summarizes recent developments in both permanent and frozen section pathology, highlights innovations such as stimulated Raman histology, and explores applications beyond gliomas, including ependymomas and primary CNS lymphomas. We discuss opportunities, limitations, and future directions for integrating AI into routine clinical practice.

## 1. Introduction

Artificial intelligence (AI) is transforming cancer pathology by addressing the global shortage of pathologists and the limitations of traditional diagnostic methods [1]. With millions of new cancer cases annually, AI—particularly deep learning (DL)—offers relatively faster and accurate analysis of the lesions, reducing variability and enhancing workflow efficiency [2]. Beyond diagnosis to offer scalable, reproducible and objective tumor classification [2], AI contributes to precision medicine by integrating histologic, genomic, and clinical data to predict treatment responses and outcomes [1,3]. These have been especially represented by successful application of AI algorithms in the management of systemic tumors including skin cancer diagnosis and the field of colonoscopy, both dependent on their peculiar “macroscopic pathology” [4,5,6,7].

There has been a huge demand for AI application for rare cancers including brain tumors. However, brain tumors have been far behind when it comes to AI development for two reasons: (1) Histologic features heavily overlap among each brain tumor type; (2) Prediction of molecular findings is essential for its typing rather than by AI-friendly macroscopic findings [8,9]. The classification of brain tumors includes a variety of histological entities, which traces its origins to the histo-genetic framework proposed by Bailey and Cushing in 1926. Further, with interobserver variability in histological approaches [10,11] as well as landmark discoveries of cardinal molecular alterations including codeletion of chromosomes 1p and 19q (1p/19q-codel) and mutations in isocitrate dehydrogenase (IDH) [12,13], the molecular classification of the brain tumors has now become the norm, represented by the 2016 revised 4th edition and the latest 5th edition of the World Health Organization (WHO) 2021 classification (WHO CNS5) [14,15,16]. Representative examples are adult diffuse gliomas which are dichotomized by IDH status, and glioblastoma is now restricted to IDH-wildtype tumors [15]. In IDH-mutant gliomas, TP53 and ATRX mutations characteristic of astrocytomas are mutually exclusive with the 1p/19q-codel that defines oligodendrogliomas [17]. Of note, malignancy or CNS WHO grading could be determined molecularly. Homozygous deletion of CDKN2A/B has been established as a marker of grade 4 IDH-mutant astrocytomas [18,19], and grade 4 glioblastoma is molecularly defined by TERTp mutation, EGFR amplification and chromosome 7+/10− [20]. The molecular framework was further advanced [21], with DNA methylation profiling incorporated for the first time as a defining criterion for certain tumor entities [22,23,24]. These advancing molecular classifications of brain tumors have made it far more complicated to develop clinically useful AI algorithms.

Despite this backdrop and formidable challenge, AI applied to hematoxylin and eosin (H&E) whole slide images (WSIs) has emerged as a promising strategy to infer molecular alterations directly from histology, offering a potential complement—or even an alternative—to traditional assays [8,9,25,26,27]. Thanks to a recent advancement in machine learning (ML) technology and multidisciplinary efforts, AI has achieved notable milestones in molecular prediction including IDH mutation, 1p/19q-codel, and MGMT methylation (prognostic/predictive epigenetic biomarker in glioblastoma), showing promise with AI-based architectures [3,28,29,30]. Despite remaining clinical challenges, the ongoing evolution toward hybrid and large-scale AI models offers a path forward, with the potential to establish AI as a clinically applicable tool for integrated diagnosis of brain tumors.

In the intraoperative setting, rapid diagnosis is equally critical for guiding surgical strategies. Frozen section-based AI approaches have assisted glioma grading [31], predicted molecular subtypes [32], and provided decision support to neurosurgeons in real time. Beyond conventional H&E sections, novel techniques such as stimulated Raman histology [33,34,35] and other label-free optical methods, when integrated with DL, are reshaping intraoperative neuropathology by producing near-real-time, high-resolution images that bypass traditional processing steps. In parallel, intraoperative nanopore sequencing has emerged as a complementary strategy, enabling rapid detection of genome-wide DNA methylation and targeted sequencing within clinically actionable timeframes [36,37,38].

Advancement in AI technology has decoded large-scale, omics datasets from H&E slides of brain tumors. The ensemble fusion framework improved prognostic/predictive accuracy beyond histology or genetics alone, highlighting the promise of combining multi-omics and clinical variables with morphology in future diagnostic pipeline. Importantly, in addition to the ML, DNA methylome-based classification of the brain tumors inferred from histopathology in brain tumors with DL [39], an omics-based model capturing the spatial transcriptome of glioblastoma demonstrated that spatial features of tumor and immune cell organization predicted patient outcomes [40]. Such notable findings underscore the potential of AI not only for classification but also for generating new biological insights into tumor microenvironmental determinants of patients’ prognosis and therapeutics prediction.

This review summarizes recent advances in AI-based neuropathology of brain tumors, with emphasis on both permanent and intraoperative applications, histology-based explainable approaches and state-of-the-art multimodal approaches targeting transcriptome and methylome profiling of brain tumors. By highlighting technical progress as well as current limitations, we aim to provide a balanced perspective on how AI is shaping the future of CNS tumor diagnosis. Representative studies in each category and representative AI studies for gliomas are depicted in Table 1 and Table 2.

## 2. Theoretical Framework for Machine Learning-Based AI Algorithm for Brain Tumors: Basics in CNNs, Transformers, and Foundation Models

The vast majority of AI approaches in computational pathology operate under the multiple instance learning (MIL) paradigm [81,82]. This framework is designed to address the core challenge of WSI analysis: models must be trained using only a single slide-level label (e.g., IDH-mutant or IDH-wildtype) for a gigapixel-scale image composed of millions of patches, or “instances.” In this weakly supervised setting, the slide is a “bag” of instances, and the model must learn to identify the salient patches that determine the bag’s label, without ever being told which specific patches are relevant. The performance of MIL framework is critically dependent on the quality of the feature representations extracted from each patch. The integration of patch-level outputs into a coherent case-level prediction remains inconsistent among models [30]. As manual annotation of millions of patches is prohibitive, the field has increasingly adopted self-supervised learning (SSL) to pre-train powerful patch-level features [83]. SSL methods learn robust, generalizable representations from massive, unlabeled pathology datasets (often encompassing millions of images across diverse cancer types), which then serve as the foundational encoders for downstream MIL tasks. Thus, the current state-of-the-art is best described as a two-stage process: patch features are first learned via SSL, and MIL aggregator (e.g., an attention-based model) is then trained on these features to perform slide-level classification. It is also notable that there is a trend moving to graph-based MIL and state space model (SSM) [84,85]. This trend closely mirrors the evolution of natural language processing (NLP) from bag-of-words to transformers and then to SSMs, reflecting a paradigm shift in computational pathology.

**Figure 1:**
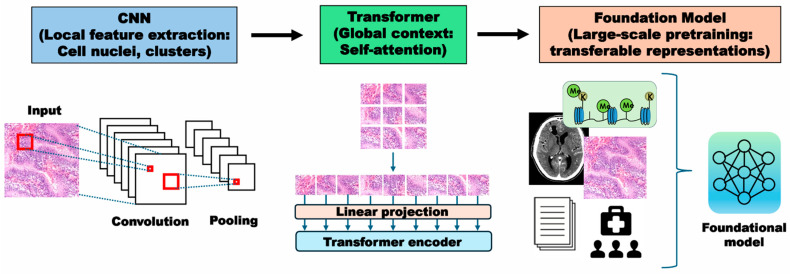
Conceptual overview of AI approaches in pathology. A schematic illustration of three major AI architectures used in brain tumor pathology: CNNs (local feature extraction), Transformer models (global context via self-attention), and Foundation models (large-scale pre-training for generalizable tasks).

The architectures predominantly used for these patch-level feature encoders are convolutional neural networks (CNNs). CNNs essentially have a series of convolution layers as the hidden deep layers, rendering them preferable at pathology image segmentation and extraction of local morphological features such as nuclear atypia and cellular clustering [86,87] (Figure 1). Using this paradigm, multiple studies have demonstrated the feasibility of predicting key glioma biomarkers. For example, Liechty et al. applied a DenseNet-based CNN to The Cancer Genome Atlas (TCGA) and a Weill-Cornell cohort, achieving an area under the curve (AUC) of 0.881 (95% confidence interval (CI)  =  0.88–0.883) for IDH mutation prediction, comparable to expert neuropathologists (0.901), and even surpassing them when combined in a hybrid human–AI workflow (0.921) (95% CI  =  0.920–0.923) [78]. *t*-test and chi-square test were used to test the difference between two IDH status groups, and CIs of model performance metrics were evaluated through sample bootstrapping for 1000 times (all statistical tests were two-sided with a significance threshold of *p*  <  0.05). Hewitt et al. implemented a multicenter weakly supervised framework on ~2845 cases to simultaneously predict IDH, ATRX, and 1p/19q status with AUC values of 0.95 in training and 0.90 in external validation for IDH, highlighting robustness across institutions [79]. As for 1p/19q-codel, an essential criterion for oligodendroglioma, Kim et al. introduced the 1p/19qNET model trained on IDH-mutant gliomas, reporting AUCs of 0.930 in a discovery cohort and 0.837 (95% CI: 0.796–0.878) in TCGA validation [80]. Remarkably, regression on copy number fold changes outperformed conventional FISH (fluorescence in situ hybridization), suggesting that CNN-based AI could provide a reliable surrogate for genetic assays. Similarly, Wang et al. demonstrated that WSIs alone were sufficient to recapitulate WHO 2021 glioma subtypes and grades, suggesting that CNN-based DL may approach the diagnostic performance of molecular assays [28].

While CNNs have remained the mainstay, their limited receptive fields constrain the modeling of long-range spatial dependencies. Transformer models, originally developed for natural language processing, address this by leveraging self-attention mechanisms to capture contextual relationships across entire WSIs [88,89] (Figure 1). In pathology AI models, CNNs are well suited for capturing local image features such as cell shapes and textures through convolutional filters clustering [86,87]. In contrast, transformer-based models focus on learning long-range relationships, allowing them to integrate global contextual information more effectively [88,89]. Regarding O6-methylguanine DNA methyltransferase (MGMT) promoter methylation, He et al. applied a Transformer-based weakly supervised model to TCGA-glioblastoma and an independent Beijing cohort, achieving AUCs of 0.86 and 0.83, with accuracies of 0.79 and 0.76, respectively–evidence of external reproducibility [69]. Of interest, in computational pathology, hybrid CNN–Transformer frameworks have already demonstrated superior performance in diffuse gliomas, especially for IDH and 1p/19q prediction [28]. Attention maps further provide interpretability, enabling pathologists to visualize regions most influential to predictions.

Beyond CNNs and Transformers, a new paradigm is emerging in the form of foundation models. These large-scale, pre-trained networks are pre-trained on millions of diverse pathology images to learn broadly transferable representations [83,90], offering improved adaptability and robustness across datasets and institutions [91,92] (Figure 1). This trend is driven by the “scaling hypothesis”–the premise that larger models trained on more extensive and diverse data will yield better performance. In brain tumor pathology, foundation models such as HIPT, Virchow [93], and UNI are being explored for molecular prediction, tumor grading, and integration of histopathology with radiology. For example, Lu et al. demonstrated the potential of pathology foundation models to generalize across cancer types [83,90], and multimodal foundation models were reported to enable fast, label-free detection of glioma infiltration, used as a general-purpose adjunct for guiding brain tumor surgeries [94]. Recent research, however, highlights a clear tension between this drive for scale and the need for specialization. Benchmarking studies suggest that model or data size does not always correlate with improved performance, particularly for highly specific or rare tasks [95,96].

Neuro-oncology, with its complex biology and reliance on uncommon molecular markers, represents a domain where generalist pan-cancer models may be suboptimal. This has fueled the development of specialized foundation models. Specialization can be achieved by focusing on a specific domain (e.g., neuro-oncology) or by integrating multiple modalities. For instance, visual-language foundation models (VLFMs) like CONCH integrate pathology reports (text) with images during pre-training [83]. This multimodal specialization allows the model to learn representations grounded in expert-derived language, significantly enhancing its utility and interpretability. Specifically, a model such as FastGlioma exemplifies a visual foundation model for brain tumors and can quickly (<10 s) detect tumor infiltration in fresh surgical tissue with high accuracy (AUC ~ 92%) [94]. Another example, DeepGlioma, uses similar principles (rapid, label-free imaging + AI) to predict molecular alterations in diffuse glioma with ~ 93% accuracy [32]. In comparison with conventional pathology/molecular diagnostics, foundational-model AI can deliver real-time, non-consumptive and scalable assessments, reducing reliance on pathologist manpower or lab infrastructure. Despite strong performance, such models may still struggle with rare tumor subtypes, extremely subtle histological/molecular features. The future of foundation models in brain tumor pathology may therefore lie not just in scaling, but in a hybrid approach: leveraging large-scale models (like CNNs and Transformers) as a base, then fine-tuning or adapting them with specialized, domain-specific data (such as brain tumor-specific images or reports) to achieve high performance on nuanced diagnostic tasks that demand expert knowledge and experience.

## 3. Deep Learning in Brain Tumor Histopathology: Updated AI Platform for CNS5-Based Genotype Prediction in Brain Tumors

### 3.1. AI Diagnostic Algorithm for FFPE-Based Permanent Sections

Gliomas, particularly diffuse gliomas, have been at the forefront of AI-driven histopathological research owing to their heterogeneous morphology and clinically relevant molecular subtypes. DL applied to formalin-fixed, paraffin-embedded (FFPE) permanent sections has enabled not only histological classification but also prediction of genetic alterations and prognosis directly from H&E-stained slides. IDH mutation status, a cornerstone of the WHO2021/CNS5, has been successfully predicted from histopathology images using AI. Liu et al. trained CNNs on FFPE slides and achieved robust accuracy in IDH prediction [41]. Extending this approach, Jiang et al. demonstrated that WSI-based models could simultaneously predict IDH mutation status and patient prognosis in lower-grade gliomas [42]. Based on these foundations, Wang et al. reported a large-scale, neuropathologist-level DL system for the integrated classification of adult-type diffuse gliomas (the difference in patient characteristics between training and the other cohorts assessed by a two-sided Wilcoxon test or Chi-square test with *p*-value  <  0.05 considered significant) [28]. In addition, Faust et al. highlighted the translational potential of AI in neuropathology, emphasizing rigorous validation, reproducibility, and integration of histology-based molecular prediction into clinical workflows [43]. Moreover, Ma et al. developed a weakly supervised pipeline, one-stop Histopathological Auxiliary System for Brain Tumors (HAS-Bt) mimicking the WHO CNS5-style classification pipeline, which expands histopathological classification to nine categories, including metastasis, lymphoma, and ependymoma in addition to glioma [44]. By utilizing slide-level predictions rather than labor-intensive pixel-level annotations, HAS-Bt achieved mean diagnostic accuracies above 90% across multiple glioma subtypes.

**Figure 2:**
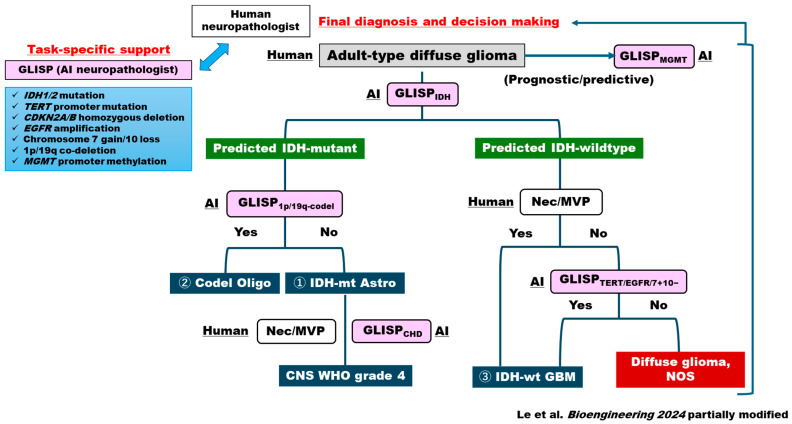
Human-AI Integrative Diagnostic Workflow based on WHO2021/CNS5 [30]. First, a pathologist examines and recognizes the morphology of gliomas in brain tumors. Next, GLISP_IDH_ classifies the tumor based on IDH status. If an IDH mutant phenotype is predicted, further molecular status testing based on the presence or absence of GLISP_1p/19q-codel_, GLISP_CHD_, microvascular proliferation (MVP), and/or necrosis enables a more molecularly integrated diagnosis compared to conventional diagnosis. If an IDH wildtype phenotype is detected, the molecular status predicted by GLISP_TERT_, GLISP_EGFR_, and GLISP_7+10−_, in addition to MVP/necrosis, is useful for further molecular diagnosis.

Recently, we further expanded the idea of AI-based prediction of genotypes for adult-type diffuse gliomas and developed an AI framework using the concept of MIL, named GLioma Image-level and Slide-level gene Predictor (GLISP) [30]. It predicts cardinal genetic/epigenetic aberration and markers of molecular grading for integrated CNS5 diagnoses in H&E sections: IDH1/2, ATRX, TP53 mutations, TERT promoter mutations, CDKN2A/B homozygous deletion (CHD), EGFR amplification (EGFRamp), 7 gain/10 loss (7+/10−), 1p/19q co-deletion, and MGMT promoter methylation [30] (Figure 2). In this study, WSIs from TCGA public data were used to train the model, validated by a total of 108 glioma cases from the Tokyo Women’s Medical University as the external dataset. Notably, the accuracy in diagnosing IDH-mutant astrocytoma, oligodendroglioma, and IDH-wildtype glioblastoma was 0.66 (95% CI = 0.56–0.74) and F1 scores for each tumor class were 0.70 (95% CI = 0.58–0.80), 0.62 (95% CI = 0.46–0.76), and 0.64 (95% CI = 0.49–0.76), respectively (sample size *N* = 108). The accuracy statistically exceeded the board-certified pathologists blinded evaluation average of 0.62 (95% CI = 0.57–0.74). GLISP thus represents a two-stage AI framework for histology-based prediction of genetic events in adult gliomas, which is helpful in providing essential information for WHO2021/CNS5 molecular diagnoses. These advances are consistent with previous reports that emphasized the promise of AI in supporting precision diagnosis in gliomas [45,46,47]. Rather unexpectedly, AI was not good at assessing the methylation status of each gene (i.e., MGMT) in comparison with genetic mutation or even genome-wide methylation profiling [30]. Further, rare tumor subtypes including pediatric-type tumors should be included in the subsequent examination. Future studies will be necessary for the human neuropathologist to exploit task-specific, flexible AI-based diagnostic algorithms to achieve reproducible, prognostic and predictive diagnostic scheme for FFPE-based brain tumor classification (Figure 2).

### 3.2. AI Diagnostic Algorithm for Intraoperative Frozen Sections

Intraoperative consultation using cryosection histology is critical for guiding neurosurgical decision-making, including assessment of tumor type, grade, and margin status. However, the diagnostic process is often challenged by technical artifacts, freezing-induced distortion, and time constraints. These limitations make cryosection histology an ideal setting for AI, where computational models can assist pathologists by enhancing accuracy and speed. Indeed, AI-assisted diagnosis in frozen sections has achieved tumor-type-dependent 85–95% accuracy in distinguishing gliomas from meningiomas, metastases, and lymphomas [34]. Notably, CNN-based models validated on large, multicenter frozen section datasets demonstrated >90% accuracy in classifying diffuse gliomas despite freezing-related artifacts [48].

An emerging direction is the intraoperative prediction of molecular alterations. Stimulated Raman histology (SRH), a label-free optical imaging technique that captures intrinsic vibrational signatures of lipids, proteins, and nucleic acids, generates high-resolution images with hematoxylin and eosin-like contrast within minutes. These images can be seamlessly analyzed by AI models to classify tumor types and predict molecular alterations [33,34]. Importantly, SRH bypasses traditional frozen-section preparation, reducing tissue loss and turnaround time (TAT), and produces standardized digital images well suited for ML pipelines [34]. Recent prospective clinical studies have demonstrated the feasibility of combining SRH with DL for real-time intraoperative decision support. Hollon and colleagues reported that an SRH-based CNN achieved non-inferior diagnostic performance compared with expert neuropathologists across more than 280 brain tumor specimens, highlighting its potential for augmenting or even substituting intraoperative pathology in resource-limited settings [34]. CNN training was replicated 10 times and the model with the highest validation accuracy was selected for use in the prospective clinical trial here, and Pearson’s correlation coefficient was used to measure linear correlations. More recent work has expanded the scope of SRH beyond morphology, enabling “virtual molecular diagnostics” whereby DL models trained on SRH images can provide predictions of clinically relevant biomarkers, including IDH mutation, 1p/19q codeletion, and ATRX status, during surgery [49,50].

Furthermore, other modalities offer non-contact, tissue-preserving advantages for intraoperative brain tumor imaging including photoacoustic remote sensing (PARS) microscope, coherent anti-Stokes Raman scattering (CARS) microscopy, confocal laser microscopy and second harmonic generation (SHG) microscopy [50,97,98,99]. In addition to optical imaging, multi-omics integration is beginning to impact intraoperative decision-making. Vermeulen et al. demonstrated that rapid single-cell and spatial profiling of brain tumors can be achieved in near-real time, opening the possibility of combining cellular architecture with AI-based predictions during surgery [36]. Building on this, Patel et al. reported that multimodal AI frameworks incorporating radiology, pathology, and genomic data improved intraoperative prediction of molecular subtype and potential therapeutic targets, supporting precision neurosurgery [37]. Most recently, Deacon et al. highlighted the feasibility of integrating AI-driven SRH with genome-wide methylation classifiers in the operating room, showing that actionable molecular insights could be generated intraoperatively with TAT compatible with surgical workflows [38]. This paradigm not only facilitates tailored surgical strategies but also illustrates the potential of combining label-free optical imaging with AI to deliver rapid, reproducible, and comprehensive intraoperative diagnostics. Nonetheless, challenges remain regarding multi-institutional validation, integration with existing workflows, and regulatory approval before widespread clinical adoption.

### 3.3. Deep Learning in Non-Glioma Primary Brain Tumors

While gliomas are the most extensively studied, AI applications in non-glioma CNS tumors are increasingly being explored. In ependymomas, morphology-based deep learning approaches have been used to support DNA methylation–defined molecular subgrouping, which is clinically relevant for risk stratification and prognosis [51,52]. AI-based epigenetic classifiers have shown potential to distinguish the challenging histological ependymoma variants such as clear cell, papillary, tanycytic and myxopallilary ependymomas as well as ZFTA fusion-positive tumors and subependymomas, complementing their molecular assays [52,53]. Schumann et al. developed deep neural network models to classifying spinal cord ependymomas into molecular subgroups, including SP-MYCN, SP-EPN, and SP-MP types, directly from routine histopathology slides [54]. For primary CNS lymphoma (PCNSL), rapid intraoperative distinction from diffuse gliomas is a major diagnostic challenge. DL models trained on multicenter frozen-section datasets have demonstrated robust performance in discriminating PCNSL, thereby facilitating timely surgical decision-making [49]. Beyond these entities, early work has extended to other tumor classes. In medulloblastomas, quantitative nuclear histomorphometry and automated image analysis have been investigated for molecular subgroup prediction (WNT, SHH, Group 3, Group 4), aligning with the WHO-integrated diagnostic framework [55,56,57]. Similarly, in meningiomas, extracted features from segmented nuclei, using a support vector machine (SVM) ensemble ML model, could classify different subtypes of meningiomas [58], and the self-organizing map (SOM) ML algorithm was reported to cluster certain features in meningioma H&E images and classify their subtypes (meningothelial, fibroblastic, transitional, psammomatous) [59]. Further, Sehring et al. developed attention-based MIL models to predict DNA methylation classes of meningiomas directly from routine H&E-stained whole-slide images [60]. Their approach achieved robust accuracy in distinguishing clinically relevant methylation subclasses and generated attention maps highlighting histologic regions linked to molecular profiles, demonstrating the feasibility of morphology-based molecular stratification [61]. Together, these findings suggest that AI can be broadly applied across CNS tumor types, offering not only morphological classification but also integrated molecular prediction. However, a caution should be made for not overestimating the reliability of intraoperative AI technology since there is currently no report on the study for time- and resource-sensitive surgery. Most studies remain in early stages with limited cohort sizes, and multi-institutional validation is essential before such approaches can be incorporated into clinical workflows [100].

## 4. The Role of Explainable AI in Neuropathology of Brain Tumors: Should AI Be Friendly to Human Neuropathologists?

A critical challenge in implementing AI for brain tumor diagnostics is the “black box” nature of DL models. While CNNs achieve high accuracy in tumor classification and molecular prediction, their decision-making processes are often opaque. This lack of interpretability raises concerns for clinical adoption, where transparency and trust are essential. Explainable AI (XAI) approaches aim to address this gap by providing visual or quantitative insights into how models derive their predictions [101,102]. In the context of neuropathology, XAI methods such as saliency maps, class activation mapping (CAM), and gradient-weighted CAM (Grad-CAM) have been used to highlight histological regions that drive classification outcomes. Heatmap visualization with DCNN of ResNet-50 demonstrated a strong ability to infer IDH status in the TCGA dataset in a weakly supervised DL-based classification for histopathology of glioma [62]. Similarly, attention-based MIL models have been successful in subtyping gliomas using pathological images, and MIL aggregation strategies (attention MIL, additive MIL) could affect the molecular prediction performance [30,63,66]. Beyond morphology, XAI has been applied to molecular prediction tasks. DL models trained to infer IDH mutation or MGMT promoter methylation from H&E slides have used feature attribution methods to identify nuclear atypia, cellular density, or vascular proliferation as predictive cues [64], and XAI can even be applied to DNA methylation-based brain tumor diagnostics [65]. Such alignment between model-derived explanations and human expertise enhances confidence in AI outputs and facilitates hypothesis generation.

Thus, explainability is not only a technical necessity but also a regulatory and ethical requirement. Transparent models are more likely to gain acceptance from both clinicians and patients, particularly in high-stakes decision-making such as intraoperative consultation or prognostic stratification [66]. However, challenges remain, and current XAI tools often provide qualitative rather than quantitative explanations, may be sensitive to image perturbations, and can produce inconsistent results across model architectures [67]. More importantly, the challenge for XAI deepens when models predict molecular features not reliably discernible by human pathologists. This phenomenon is exemplified by the prediction of microsatellite instability (MSI) in gastrointestinal cancers [103]. This finding strongly suggests that in other superhuman tasks, such as predicting IDH mutation or MGMT promoter methylation in gliomas, the models are similarly leveraging novel, sub-visual, or complex spatial features that do not map to the traditional morphological lexicon. For these tasks, XAI methodologies restricted to validating known human-defined correlates (e.g., nuclear atypia, cellular density) are fundamentally inadequate for elucidating the true basis of the model’s decision. Modern brain tumors classification totally counts on molecular genetic findings over traditional morphology due to their superior prognostic accuracy, which predict patient prognosis more accurately than morphological classification. This shift presents a paradox for XAI: approaches designed to explain AI decisions by translating them back into pathologists’ could be contradictory to genotype-based integrated diagnostic scheme since explainability namely represents morphological characteristics of the tumors in pathologists’ terms and may fundamentally conflict with a diagnostic scheme that is explicitly moving away from pure morphology. Indeed, our current attempt to exploit pathologist-friendly XAI developed for specific, morphology-based diseases such as interstitial pneumonias [68] failed to achieve higher performance than our AI framework with the use of MIL on the TCGA dataset for genotype-prediction in diffuse gliomas [Unpublished data]. We argue this “failure” is not a limitation of AI, but rather an indication that the model is likely learning novel, sub-visual, or complex spatial features lying outside the traditional morphological lexicon.

This reframes the primary role of XAI in molecular prediction: from a simple validation tool to a powerful discovery engine [101]. Instead of asking, “Did the AI find the features I already know?”, we must ask, “What new features has the AI discovered that correlate with this genotype?” By identifying these previously unknown morpho-molecular links, XAI transforms the deep learning model from an opaque “black box” into “hypothesis generator.” This discovery-oriented approach allows AI not only to assist in diagnosis but also to contribute directly to generating new biological insights, which can then be validated experimentally to advance our understanding of tumor biology. Overall, XAI represents a vital step toward clinically trustworthy, human-AI collaboration in various fields of pathology. However, its application to the integrated diagnoses of the brain tumors demands particular caution and should take a careful step in consideration. The primacy of molecular-genetic features in the CNS5-based classification scheme underscores why traditional, morphology-based explainability is insufficient. Although there has been no report to show the clear morpho-molecular relationship among brain tumors, we could cautiously embrace XAI’s potential as a discovery engine [101]—one capable of generating novel hypotheses—while rigorously validating that these new discoveries are biologically meaningful and not merely model artifacts.

## 5. Multimodal AI Platform for Integrated Diagnosis of Brain Tumors: Beyond Histo-Genetic Perspectives

Recent studies have highlighted that AI is not limited to histopathological image analysis but is also expanding into the domain of molecular neuropathology. DNA methylation profiling has emerged as a powerful tool for refining CNS tumor classification, and Capper et al. developed a random forest-based classifier trained on 2801 CNS tumors, capable of distinguishing 82 tumor classes [22]. In a prospective validation cohort of 1104 cases, the classifier agreed with histopathology in approximately 60% of samples, of which 92.8% were later confirmed by molecular analyses to favor the AI-derived classification. These findings have significantly influenced the WHO2021/CNS5 classification of CNS tumors by facilitating recognition of previously underappreciated subtypes [15,22]. The classifier has continued to evolve, with updates improving both coverage and accuracy; for example, in version V11b4 applied to 1481 CNS tumors, only 4.6% of cases were deemed completely unclassifiable (calibrated score < 0.3) [104].

Beyond array-based profiling, recent DL approaches aim to infer DNA methylation signatures, namely epigenotypes, directly from histopathology images. Hoang et al. introduced the Deploy framework, which achieved 95% overall accuracy and 91% balanced accuracy in predicting methylation-based tumor classes from H&E slides, suggesting the feasibility of bypassing separate methylation assays [39]. Similarly, weakly supervised CNNs have been developed to predict MGMT promoter methylation status from WSIs, complementing radiogenomic approaches and offering potential for integration into clinical workflows [69]. Although still in earlier stages, transcriptome-based applications also represent a promising frontier. RNA-seq and single-cell RNA-seq studies have revealed transcriptional reprogramming during glioma progression, such as the mesenchymal shift in recurrent glioblastoma, and provide granular insights into the tumor microenvironment [70,71]. Moreover, the rapid development of spatial multi-omics technology has spurred the demand for the integration of spatial transcriptomics (10× Visium, MERFISH) with DL/AI [105]. Further, future attempts should integrate proteome and metabolome information into the multimodal AI platform [72,73]. Integration of this data with AI-driven frameworks could enable more refined diagnostic and prognostic stratification, moving beyond current histo-molecular paradigms.

**Figure 3:**
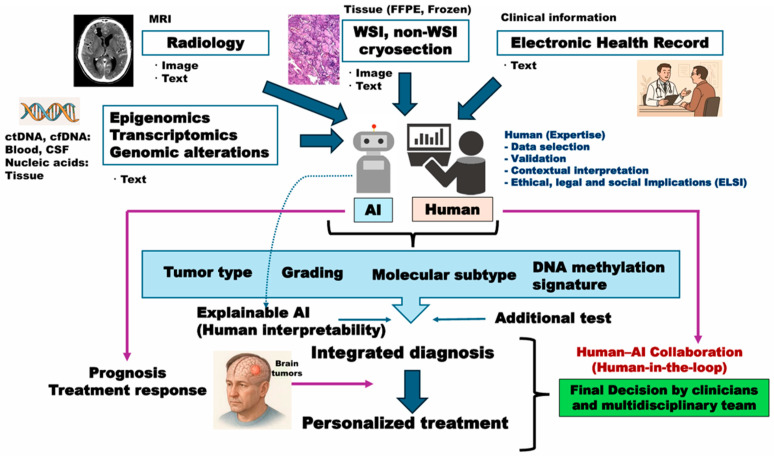
Multimodal AI framework for integrated diagnosis and precision neuro-oncology. The framework integrates complementary information used by clinicians, radiologists, and pathologists in clinical practice, represented as both imaging and text data. Radiomic features are derived from MRI scans, including AI-assisted segmentation, to capture spatial characteristics of the tumor. Surgical and biopsy specimens (FFPE and frozen) yield stained and unstained WSIs, non-WSI preparations, and cryosections. Clinical metadata (e.g., age, sex, race) from electronic health records further enrich patient profiles. For diagnostic applications, AI models combine these inputs to estimate tumor type, grade, molecular subtype, and DNA methylation class. For research purposes, liquid biopsy samples (ctDNA from blood, cfDNA from cerebrospinal fluid) and nucleic acids from tumor tissue enable epigenomic, genomic, and transcriptomic analyses, further characterizing the molecular landscape. XAI layer provides visual and quantitative verification of the model predictions. The final medical, social, and ethical responsibility remains with physicians supported by multidisciplinary teams.

Future multimodal AI frameworks should integrate radiological imaging with histopathology. By combining MRI-derived radiomic features with DL analysis of WSIs, these approaches aim to capture complementary aspects of tumor biology-macroscopic growth patterns and microscopic cellular architecture. For gliomas, multimodal models have improved the prediction of IDH mutation, 1p/19q-codel, and survival stratification, outperforming single-modality analyses [64], and even the prediction of prognosis in adult and pediatric brain tumors [74]. More recent studies have adopted attention-based and graph neural network architectures to align radiology and pathology features, demonstrating enhanced robustness and generalizability across cohorts [75,76]. These integrative AI systems are rapidly moving beyond potential and into the clinical validation phase. Recent multicenter studies on glioblastoma, for example, have demonstrated that multimodal DL models–specifically those using transformer architectures to integrate MRI-derived radiomic features, histopathology data, and clinical/molecular markers–consistently outperform unimodal models in tasks such as survival prediction [77]. This work provides concrete evidence that “radiopathomic diagnostics,” by fusing these modalities, offer more accurate and clinically actionable stratification than any single modality can alone. Despite the current limitation that no comprehensive studies have been performed to calibrate multiple heterogeneous data modalities, these advances illustrate that AI is increasingly bridging histopathology with multi-omics data including radiomics data, thereby opening the possibility of highly integrated diagnostic pipelines that combine clinical parameters, radiology, morphology, epigenetics, and transcriptional states for CNS tumor classification (Figure 3) [106].

## 6. Issues Under Active Investigation in Clinical Application of AI Models

### 6.1. H&E Variability

Current AI models in neuropathology still have several areas that require refinement before they can be reliably integrated into daily clinical practice. A primary concern involves the quality control and standardization of H&E-stained slides, which serve as the foundation for training and validating AI algorithms, as well as the WSIs generated by them. Considerable variability exists in H&E staining protocols across institutions, resulting in differences in color tone, contrast, and background clarity. These artifacts, along with fading of staining itself, can distort tissue architecture or color information and may mislead algorithms unless appropriately detected and corrected during preprocessing [107,108]. Moreover, WSI scanners from different vendors lack cross-platform compatibility, further complicating reproducibility and the generalization of trained models across institutions [109,110]. A recent study provides valuable insights for an importance of selecting appropriate DL models in achieving precise cancer classification, considering the effects of H&E stain normalization and computational resource availability, contributing to the existing knowledge on the performance, complexity, and trade-offs [111]. In data science, this variability is known as “domain shift,” a critical challenge where a model trained on data from one domain (e.g., institution A) fails to generalize to data from another domain (e.g., institution B). To address this, a primary computational approach is stain normalization. While traditional methods simply matched color statistics, recent DL-based techniques such as generative adversarial networks, or GANs, are now capable of robustly normalizing color distributions [112]. Applications of GANs are broad including virtual staining, data augmentation, domain adaptation, etc. [113]. Crucially, state-of-the-art methods are increasingly “structure-preserving,” designed to standardize color profiles while explicitly retaining the subtle morphological details and tissue architectures essential for accurate pathological diagnosis [114]. With the evaluation of a dataset containing 1420 paired H&E-stained images from two scanners, the framework achieved exceptional performance with a structural similarity index (SSIM) of 0.9663 ± 0.0076, representing 4.6% improvement over the best baseline (StainGAN), and peak signal-to-noise ratio (PSNR) reached 24.50 ± 1.57 dB, surpassing all comparison methods. An edge preservation loss of 0.0465 ± 0.0088 demonstrated a 35.6% error reduction compared to the next best method, and color transfer fidelity reached 0.8680 ± 0.0542 while maintaining superior perceptual quality. Beyond the domain adaptation methods, disruptive solutions are AI-driven virtual staining. This technique computationally generates diagnostic-quality, normalized H&E images from unstained tissue autofluorescence [115]. This approach not only bypasses the entire wet-lab chemical staining process, eliminating its variability, but also critically conserves tissue for downstream molecular assays, and there was good differentiation between tumor and nontumor regions with Dice scores above 0.8 as well as good characterization of immune cells with Dice scores of 0.85. A clinical study on lymphoma diagnostics, for example, demonstrated non-inferior diagnostic performance of virtually stained H&E images compared to conventional chemical H&E, validating the clinical feasibility of this solution [116].

### 6.2. External Validation

These technical issues are further exacerbated by the current lack of large-scale, prospective, multi-center clinical trials validating AI performance in neuropathology. Most existing studies are retrospective, relying heavily on public datasets such as TCGA, which may not reflect the diversity of staining, scanning, and clinical practices across different institutions. Therefore, rigorous external validation on multicenter, retrospective datasets is an indispensable intermediary step to mitigate bias and establish real-world generalizability, before proceeding the more complex and costly prospective trials. Without rigorous prospective validation, regulatory approval and broad clinical implementation will remain difficult [117]. A critical distinction must be made between the validation methodologies required for clinical translation. While retrospective external validation is an indispensable step to mitigate bias and establish model generalizability across diverse patient cohorts, staining protocols, and scanner types [118], it is not an ‘alternative’ to prospective validation, but rather a crucial prerequisite. External validation primarily assesses algorithmic accuracy and robustness on static datasets—a test of generalizability. In contrast, prospective validation, typically executed as a randomized controlled trial, evaluates the model’s true clinical utility and impact when integrated into a dynamic, live workflow, often in the human-in-the-loop collaboration depicted in this review (Figure 3). In addition to retrospective testing on large cohorts, most high-quality work in neuro-oncology so far is prospective non-randomized validation or external validation such as AI-assisted in situ detection of human glioma infiltration and a randomized trial with AI–detected cancer progression [119,120]. Further, consensus recommendations for standardizing brain tumor subtypes should also be important [121,122]. The current translational gap, therefore, is the profound scarcity of models that have successfully demonstrated both (1) robust performance in rigorous, multicenter external validation and (2) subsequent safety and efficacy in large-scale prospective clinical trials.

### 6.3. Digital Imaging Compatibility

Standardization efforts, such as the adoption of the Digital Imaging and Communications in Medicine (DICOM) format for WSIs, are crucial for ensuring interoperability across different platforms and institutions [123]. The lack of interoperability is being solved at an infrastructural level by the widespread adoption of the DICOM Supplement 145 standard for WSIs [124]. This enables true Vendor Neutral Archives (VNAs), allowing images from different scanners to be stored and viewed on a unified platform. Industry-wide “connectathons” have demonstrated the feasibility of this interoperability [125].

### 6.4. Other Challenges

AI design and usage have recently been closely linked to ELSI, the examination of ethical, legal, and social issues raised by the deployment of new knowledge [126]. Beyond technical considerations, there are also broader clinically relevant, ethical challenges. The so-called “black box” nature of many AI models limits interpretability and makes it difficult for pathologists and clinicians to fully trust algorithmic outputs [9]. Developing XAI frameworks that can highlight key image features influencing predictions will be crucial for clinical acceptance [101]. For clinical safety, uncertainty quantification methods (e.g., Mote Carlo dropout, Bayesian DL, ensemble approaches) should be important [127]. Equally important is establishing a consensus on ethical responsibility: When AI contributes to diagnostic or therapeutic decisions, it must be clear who bears accountability for adverse outcomes. Finally, robust safeguards for patient privacy and the security of sensitive medical data are indispensable prerequisites for the routine clinical use of AI. Harmonized guidelines for data storage, annotation, algorithm benchmarking, and reporting are equally essential. Without such frameworks, the reproducibility, transparency, and clinical reliability of AI applications in neuropathology will remain limited, and thus involvement of humans is mandatory in the future endeavor of the development and application of AI for the daily clinical practices (Figure 3). However, ELSI consideration is still on-going in the field of brain tumor pathology, and thus development of effective regulatory pathways for AI in the brain tumor pathology should be learned from the radiology/imaging field which has been taking the lead with a larger ELSI project supported by the National Institutes of Health (NIH) Brain Research through Advancing Innovative Neurotechnologies (BRAIN) Initiative [128]. Additionally, practical implementation barriers should be explored. Practical benefits and applicability of molecular testing are limited in low- and middle-income countries (LMICs) [129], and thus AI implementation may be a proposed solution for the situation. Further, implementation strategies could depend on the infrastructure. LMICs with strong foundations could favor leapfrogging strategies, while those lacking such foundations might find learning and acquisition prescriptions from absorptive capacity literature. Indeed, AI can deliver very fast, high-accuracy predictions in specific, validated workflows (e.g., SRH + CNN intraoperative diagnosis), but requires expensive hardware, broad external validation, and regulatory approval before routine replacement [34]. In contrast, for other molecular testing, immunohistochemistry (IHC) is inexpensive, fast, and accessible, but is limited when genomic detail is required. FISH remains a reliable assay for targeted copy-number calls (e.g., 1p/19q), but is slower and costlier than IHC. Next-generation sequencing (NGS) gives the most comprehensive molecular information and can be cost-effective versus serial testing, but typically has the longest TAT and requires infrastructure/pipelines [130]. Thus, one should note that there is no one-size-fits-all approach to achieving AI catch-up against current practical barriers.

## 7. Future Perspectives: Multimodal Collaboration Between Human and AI Neuropathologists

Future multimodal AI frameworks in neurooncology and neuropathology are expected to integrate radiological imaging, histopathology, omics, and clinical data into comprehensive diagnostic models (Figure 3). These approaches can capture complementary aspects of tumor biology, encompassing both macroscopic growth patterns and microscopic cellular architecture. In gliomas, such multimodal models have already improved the prediction of key molecular alterations and patient survival stratification, surpassing the accuracy of single-modality analyses [28,30]. More recent developments using attention-based and graph neural network architectures have further aligned radiology and pathology features, yielding enhanced robustness and generalizability across independent cohorts [75,131]. These advances exemplify the emerging field of multimodal diagnostics, where imaging phenotypes are systematically linked with histo-molecular signatures to enable more accurate, noninvasive, and clinically actionable tumor classification. Looking ahead, the integration of genomic data could range from mutation and copy number variations (CNVs) to higher-order genome structures including tumor-specific fragile nature of genome instability [132] and even extrachromosomal components [133]. These endeavors could facilitate highly comprehensive diagnostic pipelines for CNS tumors.

For these frameworks to achieve clinical adoption, several key challenges must be addressed. Improving interpretability remains essential: XAI techniques such as attention mechanisms, saliency mapping, and transparent graph-based reasoning should be further developed to ensure that AI-driven outputs are understandable to clinicians and pathologists. Equally important is the need for interdisciplinary collaboration—neuropathologists, radiologists, oncologists, and data scientists must work together in designing and validating these models, ensuring their clinical relevance and usability. Establishing collaborative research networks and consortia will promote knowledge sharing, accelerate validation across diverse populations, and facilitate the standardization of best practices. Finally, it is crucial to emphasize that AI should not be viewed as a replacement for morphological diagnostics, but rather as a tool to enhance them. In other word, it is not an AI but a human to be in the center of human–AI collaboration, namely “human-in-the-loop” (Figure 3). Thus, future research directions should include three perspectives: (1) Establishment of AI pathology systems should be strictly combined with multimodal approaches including radiology, molecular omics and clinical data; (2) Development of AI pathology systems should rely on two approaches of unbiased black-box vs. pathologist-friendly explainability; (3) All the diagnostic systems should be based upon human-centered approaches. When properly implemented, AI can provide quantitative evidence and decision support, ultimately improving diagnostic precision and patient care [134]. This framework is exactly applicable to the pathological diagnostic processes where it is human pathologists that make a final diagnosis of the disease with reference to AI-based datasets.

## 8. Conclusions

AI is emerging as a transformative tool in the diagnosis and management of brain tumors, with applications spanning radiology, pathology, and multi-omics. By enhancing tumor detection, classification, and characterization, AI contributes to precision medicine through improved diagnostic accuracy, personalized treatment planning, and better resource utilization. Beyond gliomas and other CNS tumors, the integration of AI into neuropathology promises to reshape clinical workflows and strengthen patient-centered care. However, realizing this potential requires overcoming persistent challenges (Table 3). Limited generalizability and interpretability could drop the performance [135,136,137,138]. Data quality, cross-platform variability, and the need for transparency in model decision-making remain critical barriers [139,140,141]. Equally important are ethical, legal, and social considerations (i.e., ELSI), including data privacy and healthcare equity, which must be addressed through robust regulatory frameworks. Continuous research, interdisciplinary collaboration, and global inclusivity in training datasets are essential to ensure generalizability and fairness, which will lead to harmonization and normalization of medicine in resource-restrained setting including LMICs [129,142]. The field is still in progress, and the majority of the successful examples here are derived from limited datasets. However, small cohort size and class imbalance may also provide methodological advantages in training pathology-based AI systems [143]. Small datasets can promote more robust feature learning when combined with self-supervised or weakly supervised approaches, which reduce reliance on spurious correlations and encourage biologically meaningful representation learning [144]. Likewise, natural class imbalance—reflecting real clinical distributions—can enhance model calibration and force algorithms to detect subtle but diagnostically relevant morphologic cues, particularly for molecular prediction tasks [81]. Further, federated learning (also known as collaborative learning), an ML technique where multiple entities collaboratively train a model while keeping their data decentralized, could be a key for privacy-preserving multi-institutional collaboration and LMIC implementation [145]. With ongoing refinement, AI-driven approaches hold great promises to predict treatment responses, improve patient outcomes, and ultimately transform the practice of neuropathology and neuro-oncology. Nevertheless, the ultimate medical, social, and ethical responsibility remains with physicians, and the rights and dignity of patients must always remain central.

## Figures and Tables

**Table 1:** Summary of representative studies in each category.

Category	Representative References
Glioma-FFPE/permanent sections	CNN in IDH prediction and patient prognosis [41,42] Neuropathologist-level DL for integrated diagnoses [28,43] Weakly supervised pipeline, HAS-Bt [44] MIL concept named GLISP [30] Perspectives on AI in supporting precision diagnosis in gliomas [45,46,47]
Glioma-frozen/intraoperative sections	Gliomas vs. meningiomas, metastases, and lymphomas [34] CNN-based models in classifying diffuse gliomas [48] Exploitation of stimulated Raman histology (SRH) [33,34] “Virtual molecular diagnostics” with SRH [49,50] Multi-omics integration in intraoperative decision-making [36,37,38]
Non-glioma primary brain tumors	Ependymomas with genotype/risk stratification and prognosis [51,52,53,54] Primary CNS lymphoma (PCNSL) with surgical decision-making [49] Medulloblastomas with nuclear histomorphometry and molecular subgroup [55,56,57] Meningiomas using a SVM ensemble ML model and the SOM ML algorithm [58,59] as well as prediction of DNA methylation classes for meningiomas [60,61]
Explainable AI models	Saliency maps, CAM, grad-CAM, and heatmap to infer IDH status in the TCGA dataset [62] Attention-based MIL models in subtyping gliomas [63] Histologic parameters to infer IDH mutation or epigenotypes (methylome and MGMT status) [64,65] Technical challenges and human biases [66,67,68]
Multimodal AI models	DL approaches for epigenotyping into clinical workflows [39,69] Transcriptome-based applications during glioma progression [70,71] Integrated proteome and metabolome approaches [72,73] Integration of radiomic features with histo-molecular and clinical data (radiopathomic) [64,74,75,76,77]

**Table 2:** Representative studies of AI-based pathology diagnostic systems for gliomas.

Citation (First Author, Year)	Task/Target	Architecture (Representative)	Dataset & Cohort Size	Protocol (Preproc/Training)	Validation Approach	Reported Performance
Liechty, 2022 (Sci Rep) [78]	IDH mutation prediction from H&E WSI	Multi-scale ResNet patch classifiers ensemble + slide aggregation (multi-magnification ensemble)	TCGA + institutional cases; total ≈ 500 slides	WSI tiling at multiple magnifications (2.5×, 10×, 20×); patch CNN training; ensemble across scales; pathologist–model fusion experiments	Train/val/test with external institutional test; comparison with neuropathologists; bootstrapped CIs	ML max AUC ≈ 0.88 (human AUC ≈ 0.90); hybrid human + ML AUC ≈ 0.92
Hewitt, 2023 (Neurooncol Adv) [79]	Direct image → WHO subtyping (IDH/ATRX/1p19q, etc)	Weakly supervised MIL + transformer attention	Multi-center cohorts, N = 2845 patients (multiple tumor types)	Slide-level weak supervision (MIL), patch sampling, stain normalization	External validation across multiple cohorts; held-out test sets	Training AUROCs (IDH 0.95; ATRX 0.90; 1p/19q 0.80); External AUCs: IDH ~0.90, ATRX ~0.79, 1p/19q ~0.87
Kim, 2023 (NPJ Precis Onc) [80]	1p/19q codeletion (IDH-mutant gliomas)	Weakly-supervised slide-level network “1p/19qNET” (patch CNN + regression head)	Discovery DS N = 288; external IVS (TCGA) N = 385	Slide tiling, weak labels from NGS/FISH; trained to predict fold-change per arm; explainable heatmaps	Cross-validation on DS; external validation on TCGA	R^2^ (1p) = 0.589, R^2^ (19q) = 0.547; AUC (IDH-mutant classifier) DS 0.93, IVS 0.837
Wang, 2023 (Nat Commun) [28]	Integrated WHO-style classification from H&E WSIs (adult diffuse gliomas)	Multi-scale MIL + ResNet encoders; slide-level integrated decision pipeline	Training n = 1362; validation n = 340; internal test n = 289; 2 external test cohorts n = 305 & 328	Multi-scale patch extraction, MIL pooling, integrated outputs for type/grade/genotype	Internal + two external cohorts (multi-center)	High performance; AUROC > 0.90 for major tumor types and genotype classification; subtype accuracy > 90%
Ma, 2023 (J Neurooncol)—HAS-Bt [44]	WHO-CNS5 style multi-task pipeline for histopathologic diagnosis	Pipeline MIL (pMIL) with patch encoder + decision logic	1038 slides; 1,385,163 patches for training; independent test 268 slides	Patch extraction, pMIL pipeline, built-in decision tree using molecular markers when available	Internal train/val + independent test set	9-class classification accuracy 0.94 on independent dataset; processing time ~443 s/slide
Le (GLISP), 2024 (Bioengineering) [30]	Multi-gene predictors (IDH, ATRX, TP53, TERTp, CDKN2A/B, EGFRamp, 7+/10−, 1p/19q, MGMT) from H&E	Two-stage GLISP: patch-level GLISP-P + slide-level GLISP-W (MIL-like)	TCGA training; external Tokyo Women’s Medical Univ external set n = 108	Patch CNNs, two-stage aggregation (patch → slide), gene-specific output heads	Cross-validation + external Tokyo Women’s Medical Univ testing	Patch/case AUCs: IDH1/2 ~0.75/0.79; 1p/19q patch/case ~0.73/0.80; overall diagnosis accuracy 0.66 (exceeds human avg 0.62)
Hollon et al., 2020 (Nat Med) [34]	Near-real-time intraop diagnosis using Stimulated Raman Histology (SRH) + DL	CNN (“SRH-Net”) trained on SRH tiles; rapid inference pipeline	>2.5 million SRH images aggregated across studies; clinical trials: 278 patients across 3 hospitals	SRH imaging of fresh tissue intraop; CNN tile classifier; slide-level aggregation; prospective real-time pipeline (~150 s)	Prospective trials across hospitals; comparison to frozen section and final diagnosis	Diagnostic accuracy 94.6% (rapid SRH + DL) vs. 93.9% conventional methods; real-time capability
Hollon et al., 2023 (Nat Med) [32]	Label-free optical imaging (SRH) → molecular classification of diffuse gliomas	CNN classifier on SRH images; optical image → molecular label pipeline	Single/multi-center SRH datasets; ~150 glioma cases in reported prospective evaluation	SRH acquisition (fresh tissue), CNN training on SRH tiles with molecular labels; per-tile → slide aggregation	Prospective evaluation; clinical intraoperative settings	Reported molecular-class prediction accuracies ~90% in prospective setting
Patel et al., 2025 (Nat Med) [37]	Prospective multicenter validation of rapid molecular profiling (Rapid-CNS^2^)	Integrated nanopore sequencing + methylation classifier (MNP-Flex) + ML methylation classifier	Validation cohort = 301 archival + prospective samples (including 18 intraop) + global classifier validation cohort > 78,000 samples for MNP-Flex	Adaptive sampling nanopore sequencing intraop (real-time methylation + CNV), MNP-Flex classifier trained on multi-platform methylation data	Prospective multicenter validation, intraoperative runs	MNP-Flex: 99.6% accuracy for methylation families; Rapid-CNS^2^ provides real-time methylation classification within 30 min and full profile within 24 h
Hoang et al., 2024 (Nat Med) [39]	Predict DNA methylation–defined CNS tumor types from histopathology (DEPLOY/related)	Deep ensemble: direct model + indirect (predict beta values) + demographic model; combination (DEPLOY)	Internal training n = 1796; external test datasets combined n = 2156; total multi-center > 3900	Patch CNN encoders; predict methylation beta values then classify; high-confidence filtering	Three independent external test datasets (multicenter)	Overall accuracy 95% and balanced accuracy 91% on high-confidence predictions (ten-class mapping)
Deacon et al., 2025 (Neuro Oncol)—ROBIN [38]	Ultra-rapid nanopore assay (ROBIN) integrating intraop methylome classification + next-day profiling	Nanopore signal classifier + methylation ML pipeline	Prospective intraop series: 50 cases (reported) in initial evaluation; larger multicenter described	Rapid library prep + nanopore run; three methylation classifiers operating in pipeline; live classification within minutes	Prospective evaluation (intraop)	Concordance with final integrated diagnosis ≈ 90% in prospective set; turnaround < 2 h for intraop classification

**Table 3:** Key challenges and proposed solutions in AI-based brain tumor diagnostics.

Challenge	Key Issues	Proposed Solutions
Limited generalizability	Performance drops across scanners, institutions, and patient populations due to domain shift	Large, multi-institutional datasets (TCIA, BraTS, federated consortia, etc) [135] Generalizable Pathology Foundation Model [136]
Data quality & label noise	Variability, artifacts, and inconsistent annotations reduce model reliability	Rigorous imaging QC pipelines [139,140]
Small cohorts & class imbalance	Rare tumor subtypes lead to insufficient training data and biased model calibration	Oversampling, focal loss, class-balanced loss [143]
Lack of biological interpretability	Limited clinical trust due to “black-box” predictions without biological rationale	Attention maps, saliency methods [138] Pathway-guided interpretable DL architectures [137]
Limited prospective/clinical validation	Most models remain retrospective; few prospective or real-world evaluations exist	Prospective multi-site validation [37,100]
Reproducibility & transparency	Limited public code/model reporting reduces trust and regulatory readiness	Open-source pipelines (MONAI, nnU-Net) [141] Federated learning [145]
